# Quantifying Leg Muscle Disuse Atrophy During Bed Rest Using DXA, CT, and MRI

**DOI:** 10.1002/ejsc.12299

**Published:** 2025-04-10

**Authors:** Cas J. Fuchs, Wesley J. H. Hermans, Job van den Hurk, Christopher J. Wiggins, Per Widholm, Olof Dahlqvist Leinhard, Pandichelvam Veeraiah, Joachim E. Wildberger, Jeanine J. Prompers, Luc J. C. van Loon

**Affiliations:** ^1^ Department of Human Biology NUTRIM Institute of Nutrition and Translational Research in Metabolism Maastricht University Medical Centre^+^ Maastricht the Netherlands; ^2^ Scannexus Ultra High‐Field MRI Center Maastricht the Netherlands; ^3^ AMRA Medical AB Linköping Sweden; ^4^ Department of Radiology and Nuclear Medicine School for Cardiovascular Diseases (CARIM) Maastricht University Medical Centre^+^ Maastricht the Netherlands

**Keywords:** computed tomography, dual‐energy x‐ray absorptiometry, magnetic resonance imaging, muscle mass, muscle volume, skeletal muscle

## Abstract

This study evaluated whether dual‐energy X‐ray absorptiometry (DXA), computed tomography (CT), and magnetic resonance imaging (MRI) provide comparable outcomes in quantifying disuse‐induced skeletal muscle atrophy. Although the calculation of muscle volume using MRI analysis may be considered the gold standard, the method remains labor intense and, as such, less practical and more costly. In this context, we also evaluated the efficacy of a commercially available automated MRI analysis method to measure changes in leg muscle volume after two weeks of bed rest. Twelve healthy, male adults (age: 24 ± 3 years, BMI: 23.7 ± 3.1 kg/m^2^) were subjected to 2 weeks of strict bed rest. Leg muscle assessments were performed before and after bed rest using DXA, single slice (thigh) CT, and MRI. MRI data analyses were performed using both a manual and automated (AMRA) method. Leg lean mass, as assessed with DXA, declined by 5% following bed rest (from 10.2 ± 1.6 to 9.7 ± 1.6 kg; *p* < 0.001). The thigh muscle cross‐sectional area, as assessed with CT, declined by 6% following bed rest (from 155 ± 26 to 146 ± 24 cm^2^; *p* < 0.001). Muscle volume, as assessed using MRI, declined by 5% following bed rest, both when assessed manually (from 7.1 ± 1.1 to 6.7 ± 1.0 L; *p* < 0.001) and automatically (from 7.2 ± 1.1 to 6.8 ± 1.0 L; *p* < 0.001). A very strong correlation (*r* = 0.96; *p* < 0.001) with a low bias (−0.11 ± 0.29 L) was observed between manual and automated muscle volume analysis. DXA, CT, and MRI all show a ∼5% decline in leg muscle quantity following two weeks of bed rest in healthy adults. When using MRI, disuse muscle atrophy can be accurately quantified using an automated approach, rendering time‐consuming manual analysis obsolete.

**Summary**

- DXA, CT, and MRI detect a similar decline in leg muscle quantity following two weeks of bed rest in healthy adults.
- Automated MRI analysis accurately assesses bed rest‐induced muscle atrophy, offering a time‐efficient alternative to traditional manual MRI methods.
- Muscle atrophy during bed rest is more pronounced in the anterior thigh compartment compared to the posterior compartment, highlighting muscle group‐specific responses to disuse.

**Abbreviations**
CI: confidence interval
CSA: cross‐sectional area
CT: computed tomography
CV: coefficient of variation
DXA: dual‐energy X‐ray absorptiometry
ICC: intraclass correlation coefficient
MRI: magnetic resonance imaging

## 1 Introduction

Skeletal muscle, accounting for 40%–50% of body mass, is essential for vital functions such as respiration, movement, posture, and metabolism (Frontera and Ochala 2015). Muscle mass declines significantly during periods of inactivity or bed rest, often due to sickness or surgery, and such periods are thought to play a key role in age‐related sarcopenia (English and Paddon‐Jones 2010; Wall and van Loon 2013; Oikawa et al. 2019). This loss of skeletal muscle mass often predisposes to complications and increased morbidity and mortality (Lee et al. 2021; Zhou et al. 2023; Lopez et al. 2019; Prado et al. 2018). Therefore, it is critical to study the impact of physical inactivity and bed rest on muscle atrophy using appropriate methodologies that can accurately quantify this decline. Several imaging techniques are available for assessing muscle atrophy during bed rest (Rodriguez et al. 2024), with magnetic resonance imaging (MRI) considered the gold standard for skeletal muscle mass quantification (Mitsiopoulos et al. 1998). Ultrasonography is another widely applied technique and has previously been validated against MRI during disuse (Stokes et al. 2021) and bed rest (Arbeille et al. 2009; Scott et al. 2017). Other frequently applied imaging modalities to assess muscle mass and atrophy in both clinical and research settings are dual‐energy X‐ray absorptiometry (DXA) and computed tomography (CT) (Hodgson et al. 2022; Marusic et al. 2021; Dirks et al. 2018; Cruz‐Jentoft et al. 2010; Nikodinovska and Ivanoski 2023; Sabatino et al. 2020). Therefore, it is important to compare DXA and CT with MRI to evaluate their effectiveness in quantifying muscle loss induced by bed rest.

DXA is a two‐dimensional imaging technique that distinguishes between bone and soft tissue using X‐rays with two different energies. This allows for precise measurement of bone mineral density (Morgan and Prater 2017; Garg and Kharb 2013) and gives estimations of fat mass and lean mass, which often is used as a surrogate for muscle mass. Although DXA is generally a cost‐effective, safe, and rapid method to provide detailed body composition analysis (Shepherd et al. 2017; Nana et al. 2015), its two‐dimensional nature limits direct volumetric measurements of specific compartments, such as specific (e.g., quadriceps) muscle groups. Gaining more detailed insights into changes in leg muscle mass is, however, important, given its impact on functional capacity (Lindemann et al. 2016; Patiño‐Villada et al. 2020). As an alternative imaging technique, CT could provide high‐resolution three‐dimensional images of the body by scanning over a minimum angular range of 180° plus fan angle (Kalender 2011). Theoretically, this does allow for direct volumetric measurements of specific muscle groups. In practice, however, CT is often restricted to two‐dimensional analyses of one or a few axial slices to minimize ionizing radiation exposure (Yu et al. 2009; Bos et al. 2023). As a result, the single‐slice CT of the upper leg is often applied, thereby lacking a comprehensive volumetric assessment of the total leg muscle. MRI utilizes unique magnetic properties of hydrogen nuclei in water and fat to generate detailed images of soft tissues. This enables a precise quantification of muscle volume. The advantage of MRI is that it allows for complete volumetric imaging without using ionizing radiation. Moreover, this also allows the quantification of muscle volumes of distinct areas, offering more detailed insights into regional muscle atrophy (Bass et al. 2021; Kilroe et al. 2020; Fuchs et al. 2023; Maden‐Wilkinson et al. 2013). MRI has demonstrated excellent reproducibility (global reproducibility of 1.1%) for quantifying regional muscle volumes, such as the quadriceps (Nordez et al. 2009).

Although MRI, given its ability to provide more detailed insights into muscle atrophy, can be considered more favorable compared to DXA and CT (Fuchs et al. 2023; Maden‐Wilkinson et al. 2013), it also comes with significant drawbacks. Besides the costs, required infrastructure, and specialized personnel, the actual data analysis to reconstruct and calculate muscle volumes is time‐consuming. We have previously shown that the manual assessment of total leg muscle volume (including individual muscle groups) can take up to 10 working days, full‐time, for one individual (Fuchs et al. 2023). To overcome this issue, the last decade has seen significant advancements and enhancements in (semi‐) automated (commercially or publicly available) assessment methods. Examples include, but are not limited to, SliceOmatic (Tomovision, Quebec, Canada), Springbok Analytics (Charlottesville, VA, USA), Dafne (Deep Anatomical Federation Network; open‐source software platform), 3D slicer (open‐source software platform), Materialise Mimics (Materialise NV, Leuven, Belgium), and AMRA researcher (AMRA Medical AB, Linköping, Sweden). AMRA researcher, in particular, also use image calibration techniques that yield consistent results across different scanner models and field strengths (Borga et al. 2020). If such an automated system, which significantly expedites data processing and enhances feasibility, is able to maintain high accuracy in detecting small differences in muscle disuse atrophy, it would be deemed suitable for (large‐scale) clinical intervention trials and render time‐consuming manual analysis obsolete.

In the present study, we evaluated whether DXA, CT, and MRI provide comparable outcomes in assessing leg muscle atrophy in vivo in humans after 2 weeks of strict bed rest. In addition, we compared and evaluated the correlation and agreement between manual MRI analysis and a, commercially available, automated MRI analysis method.

## 2 Materials and Methods

### 2.1 Participants

The data for this investigation were derived from a randomized controlled trial conducted in our lab (Fuchs, Hermans, et al. 2024). Twelve healthy young men (age: 24 ± 3 years; body mass: 79.4 ± 12.4 kg; height: 1.83 ± 0.07 m; BMI: 23.7 ± 3.1 kg/m^2^) were included in the current study. All participants were informed on the nature and risks of the experiment before written informed consent was obtained. The current study was approved by the Medical Ethical Committee of Maastricht University Medical Center (registration number 17‐3‐014) in accordance with the Declaration of Helsinki. This trial was registered at the former Dutch trialregister.nl as with the number of NL6222. This is now available via <https://www.onderzoekmetmensen.nl/en/trial/22281>.

### 2.2 General Study Design

In the original study, we examined the effects of daily unilateral blood flow restriction on leg muscle atrophy during a 2‐week strict bed rest period. Given that we did not observe any effect of blood flow restriction on any leg muscle assessment (i.e., DXA, CT, and MRI) (Fuchs, Hermans, et al. 2024), we utilized data from both legs to explore the impact of various measurement techniques on leg skeletal muscle atrophy during bed rest.

The complete experimental design and procedures are extensively described in our recently published article (Fuchs, Hermans, et al. 2024). In short, all participants were subjected to 2 weeks of strict bed rest and were not permitted to leave the bed for any reason, with all hygiene and sanitary activities performed while lying in bed. Before and after 2 weeks of bed rest, following overnight fast, DXA, single slice (thigh) CT, and MRI were performed on both legs. Before all scanning procedures, participants were asked to empty their bladder and bowels (which was performed in bed at the end of the bed rest period in order for participants to remain inactive). During the 14‐day bed rest period, food was carefully controlled, standardized, and provided regularly (breakfast, lunch, dinner, and snacks in between). For more detailed information, please refer to our recently published article (Fuchs, Hermans, et al. 2024). On the final day of bed rest, participants were transported in a wheelchair to their DXA, CT, and MRI scans to maintain inactivity.

### 2.3 Scanning Procedures

#### 2.3.1 DXA

The leg lean mass of both legs was measured using DXA (Hologic, Discovery A; QDR Series, Marlborough, MA, USA). The system's software package Apex version 4.0.2 was used. All DXA scans and leg lean mass analyses were performed on the same system by the same technician. Each participant underwent scanning in a supine position with knees and hips fully extended and the arms resting next to the trunk with the hands in a pronated position. Leg lean mass was determined by applying a border through the femoral neck. The coefficient of variation (CV) for repeated scans to assess leg lean mass was 1.3% (Fuchs et al. 2023).

#### 2.3.2 CT

The anatomical cross‐sectional area (CSA) of the thigh muscle of both legs was assessed via a single‐slice CT scan on a second generation dual‐source CT (Somatom Definition Flash; Siemens Healthineers, Forchheim, Germany). While participants were lying supine, with their hips and knees fully extended and their feet secured, a 2‐mm thick axial image was taken 15 cm proximal to the top of the patella. The precise scanning position was marked with a semipermanent ink for replication after the 14‐day bed rest period. The following scanning characteristics were used: 120 kV, 300 mA, rotation time of 1 s, and a field of view of 500 mm. Tissue with Hounsfield units between −29 and 150 HU was selected as muscle tissue. All CT analyses were performed blinded by the same investigator (CV: 0.6% (Fuchs et al. 2023)). CT scans were analyzed for the CSA of the thigh muscles by manual tracing using ImageJ software (version 2.0.0; National Institutes of Health, Bethesda, MD, USA).

#### 2.3.3 MRI

MRI images were obtained at Scannexus (Brightlands Maastricht Health Campus, Maastricht, the Netherlands). All MRI scans were performed by the same technician. Participants were scanned in the supine position (entering the magnet head first), with knees and hips fully extended, on a 3T system (Magnetom Prisma Fit, Healthineers, Erlangen, Germany) using a whole‐body coil (Siemens). Using standard 3D 2‐point Dixon Vibe sequences, water‐ and fat‐separated volumetric data with neck‐to‐knee coverage were acquired, with a total scan‐time of 6 min. Scanning parameters were flip angle (α) = 10°, repetition time (TR) = 3.89 ms, echo time (TE) = 1.22/2.45 ms, and bandwidth = 930 Hz/Px with a 256 × 192 matrix. The slabs covering the thighs consisted of 88 slices with a voxel size of 2.0 × 2.0 × 3.0 mm^3^ and were acquired during free breathing. Muscle volume was analyzed for the entire thigh (including some pelvic) muscle groups combined (i.e., obturator externus, adductor muscle groups, iliopsoas, gluteus muscle group, hamstring muscle group, quadriceps femoris muscle group, sartorius, and tensor fasciae latae) of both legs. In addition, we separately analyzed both the anterior (i.e., quadriceps femoris muscle group, sartorius, and tensor fasciae latae) and posterior (i.e., obturator externus, adductor muscle groups, iliopsoas, gluteus muscle group, and hamstring muscle group) muscle groups of both legs.

**FIGURE 1:**
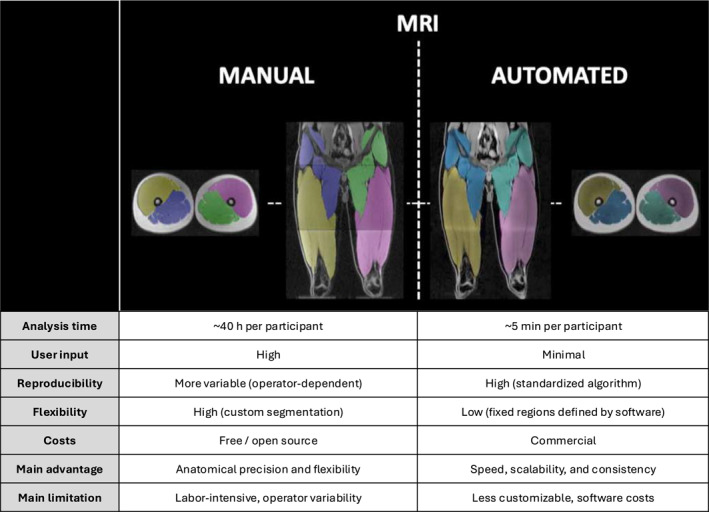
Transverse/axial and coronal images of the manual (left) and automated (right) MRI segmentation techniques. For manual segmentation, MRI images were segmented and colored manually using ITK‐SNAP software. For automated segmentation, MRI images were processed using AMRA Researcher (AMRA Medical AB, Linköping, Sweden), which applies a nonrigid multi‐atlas segmentation technique to automatically analyze thigh muscle volume, with manual quality control. Both methods included segmentation of the anterior and posterior muscle compartments. A summary table highlighting the key differences between manual and automated analysis is included directly below for reference.

We used the same images for both manual and automatic segmentation analysis (Figure 1). Manual muscle volume analysis was performed using ITK‐SNAP software (Yushkevich et al. 2006), as described previously (Fuchs et al. 2023). For automated segmentation analysis, we exported the dataset to a commercially available body composition tool (AMRA Researcher, AMRA Medical AB, Linköping, Sweden) to automatically analyze thigh muscle volume (including separation between the anterior and posterior muscle volume), as also described in detail previously (Karlsson et al. 2015; Thomas et al. 2014; West et al. 2018; Fuchs, Trommelen, et al. 2024). In short, AMRA Researcher uses advanced image processing algorithms to provide rapid, automated segmentation, and quantification of muscle volume, utilizing a nonrigid multi‐atlas segmentation technique together with manual quality control. The assessors were blinded to participants and measurement times during the analysis. In addition, manual and automated MRI analyses were conducted independently and blinded to each other's results prior to comparison.

### 2.4 Statistical Analysis

Given that the initial muscle mass of the left and right legs was equivalent, and both legs experienced the same level of muscle atrophy, as confirmed by all imaging techniques (DXA, CT, and MRI) (Fuchs, Hermans, et al. 2024), we took the average value of the left and right leg for the assessment of leg muscle atrophy. This provided 12 data points for comparison. For the comparative analysis of imaging techniques and to evaluate automated versus manual MRI analysis, the left and right legs were considered independent of each other, providing 24 data points for each time point (pre‐ and post‐bed rest). Data in the text are presented as mean ± standard deviations, and in figures as mean values with individual data points, unless stated otherwise. Normality was confirmed with the Shapiro–Wilk test. Paired sample *t*‐tests were performed to assess changes before and after 2 weeks of strict bed rest (when assessed with each imaging technique) and to compare the magnitude of atrophy between manual and automated MRI analysis. The association between imaging techniques and the comparison of automated versus manual MRI analyses were examined through linear regression, with Pearson's *r* used to assess the correlation. Additionally, agreement between MRI analysis methods was assessed using Bland–Altman plots, detailing mean bias ± SD and 95% limits of agreement. The two‐way absolute intraclass correlation coefficient (ICC) with a 95% confidence interval (CI) was also calculated to evaluate agreement between the manual and automated MRI analyses. The coefficient of variation (CV) with a 95% CI was calculated to quantify variability. The alpha level for statistical significance was set at 0.05. Analyses were conducted using SPSS (version 26.0, IBM Corp., Armonk, NY, USA).

## 3 Results

### 3.1 DXA

**FIGURE 2:**
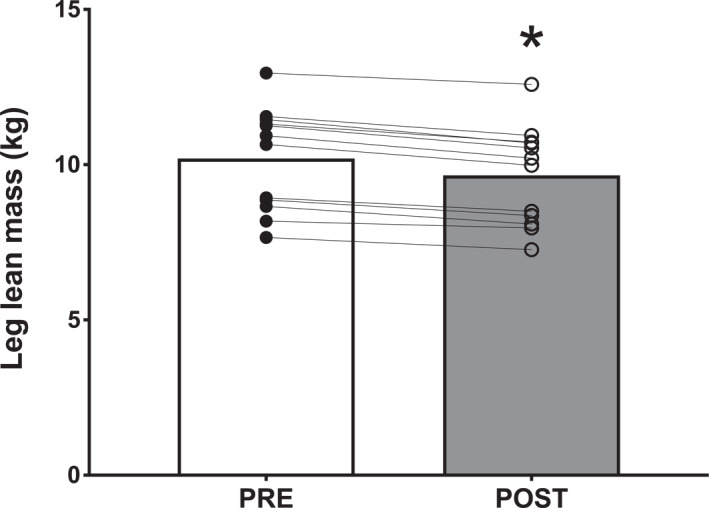
DXA‐derived leg lean mass before (PRE) and after (POST) 2 weeks of strict bed rest in healthy, young men (*n* = 12). The average of the left and right leg was taken for comparing pre‐ and post‐bed rest. Bars are means and dots represent individual values. *, significantly different (*p* < 0.001) from PRE.

DXA‐derived leg lean mass declined by 5% (546 ± 165 g) following bed rest, from 10.2 ± 1.6 to 9.7 ± 1.6 kg (*p* < 0.001; Figure 2).

### 3.2 CT

**FIGURE 3:**
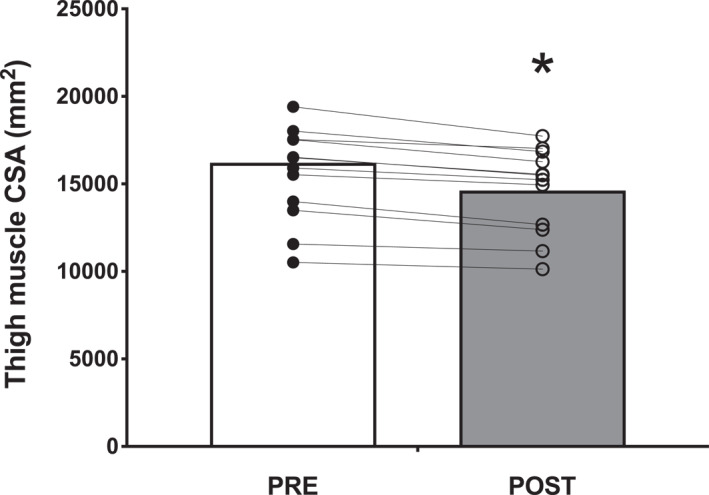
CT‐derived thigh muscle CSA before (PRE) and after (POST) 2 weeks of strict bed rest in healthy, young men (*n* = 12). The average of the left and right leg was taken for comparing pre‐ and post‐bed rest. Bars are means and dots represent individual values. CSA, cross‐sectional area. *, significantly different (*p* < 0.001) from PRE.

The CT‐derived thigh muscle cross‐sectional area declined by 6% (9.2 ± 4.1 cm^2^) following bed rest, from 155 ± 26 to 146 ± 24 cm^2^ (*p* < 0.001; Figure 3).

### 3.3 MRI

#### 3.3.1 Total Thigh Muscle Volume

**FIGURE 4:**
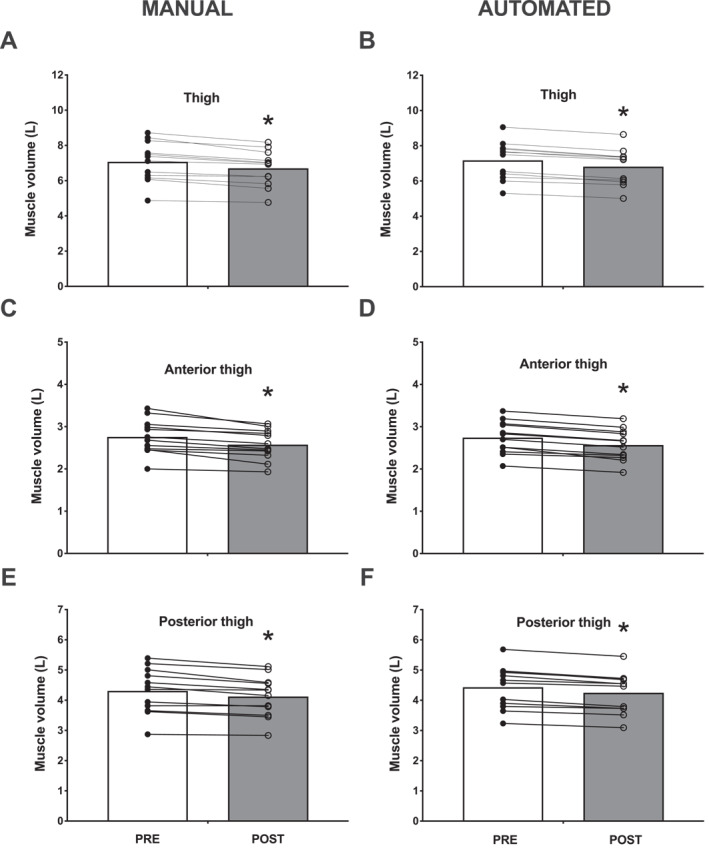
MRI‐derived total thigh muscle volume (manual assessment (A) and automated assessment (B)), anterior thigh muscle volume (manual assessment (C) and automated assessment (D)), and posterior thigh muscle volume (manual assessment (E) and automated assessment (F)) before (PRE) and after (POST) 2 weeks of strict bed rest in healthy, young men (*n* = 12). The average of the left and right leg was taken for comparing pre‐ and post‐bed rest. Bars are means and dots represent individual values. *, significantly different (*p* < 0.001) from PRE.

When assessed manually, MRI‐derived thigh muscle volume declined by 5% (372 ± 214 mL) following bed rest, from 7.1 ± 1.1 to 6.7 ± 1.0 L (*p* < 0.001; Figure 4A). When assessed automatically, MRI‐derived thigh muscle volume also showed a decline by 5% (360 ± 108 mL) following bed rest, from 7.2 ± 1.1 to 6.8 ± 1.0 L (*p* < 0.001; Figure 4B). The magnitude of atrophy did not differ between the two methods (*p* = 0.920). Marginally higher values (1.8 ± 4.1%) of thigh muscle volume were observed with automated versus manual analyses for both the pre‐ and post‐bed rest values.

**FIGURE 5:**
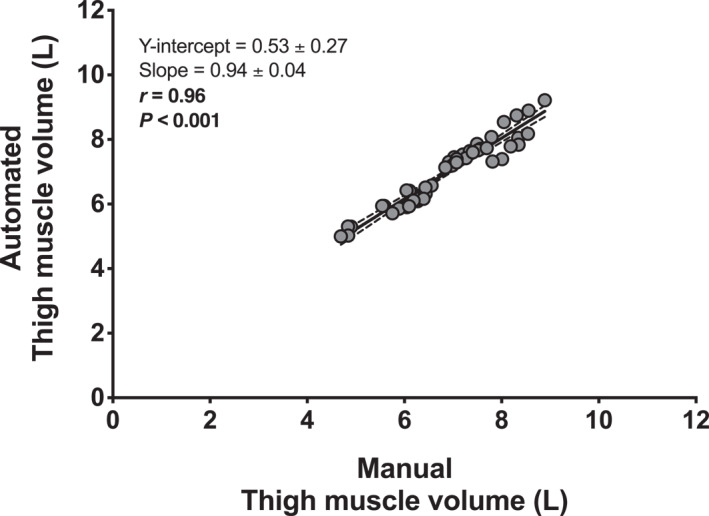
Scatter plots for the comparison between the automated and manual assessment of MRI‐derived total thigh muscle volume from both legs of healthy, young men (*n* = 12) before and after a 2‐week bed rest period. The best fit line with 95% confidence bands together with the *Y*‐intercept, slope, *r*, and *p* values are provided in the figure.

**FIGURE 6:**
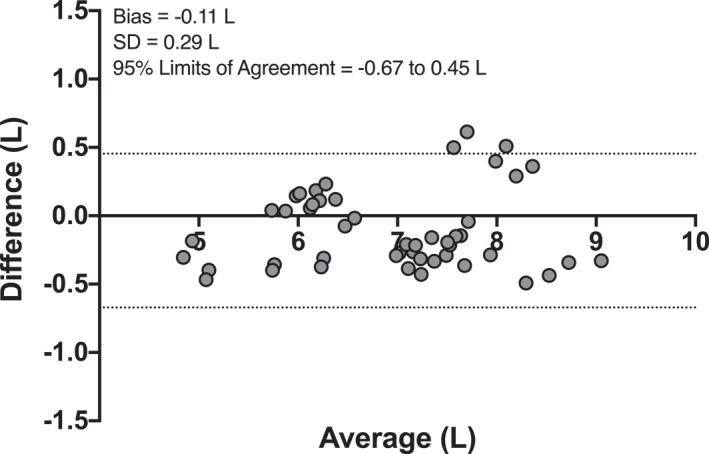
Bland–Altman plot illustrating the agreement between automated and manual assessment of MRI‐derived total thigh muscle volume from both legs of healthy, young men (*n* = 12) before and after a 2‐week bed rest period. The plot shows the differences between the automated and manual measurements against the averages of these two methods. The mean difference (Bias), standard deviation (SD) of the differences, and the 95% limits of agreement (calculated as the mean difference ± 1.96 × SD) are displayed. The upper and lower limits of agreement are highlighted, providing visual insights into the consistency of the two assessment methods across the range of measurements.

A very strong correlation was observed between the manual and automated assessment of thigh muscle volume (*r* ^*2*^ = 0.93, *r* = 0.96, 95% CI: 0.93 to 0.98; Figure 5). Also, when the legs (left and right) and time points (pre and post bed rest) were assessed separately (12 data points for comparison), we found similar correlations (*r* = 0.95–0.97; *p* < 0.001 for all). In addition, a very good agreement was observed between both analysis methods, as assessed with the Bland–Altman plot, with a low bias (−0.11 ± 0.29 L) and 95% limits of agreement being between −0.67 and 0.45 L (Figure 6). The ICC between the manual and automated analyses was 0.96 (95% CI: 0.92–0.98), with a CV of 2.74 (95% CI: 2.32–3.17) %.

#### 3.3.2 Anterior Thigh Muscle Volume

When assessed manually, MRI‐derived anterior thigh muscle volume declined by 7% (183 ± 112 mL) following bed rest, from 2.8 ± 0.4 to 2.6 ± 0.4 L (*p* < 0.001; Figure 4C). When assessed automatically, MRI‐derived anterior thigh muscle volume showed a similar decline of 6% (177 ± 58 mL) following bed rest, from 2.7 ± 0.4 to 2.6 ± 0.4 L (*p* < 0.001; Figure 4D). The magnitude of atrophy did not differ between the two methods (*p* = 0.974) and only marginally lower values (0.4 ± 3.7%) of anterior thigh muscle volume were observed with automated versus manual analyses for both the pre‐ and post‐bed rest values.

A very strong correlation was observed between the manual and automated determination of anterior thigh muscle volume (*r* ^*2*^ = 0.92, *r* = 0.96, 95% CI: 0.93 to 0.98, *p* < 0.001). In addition, a very good agreement was observed between both analysis methods, as assessed with the Bland–Altman plot, with a very low bias (0.01 ± 0.11 L) and 95% limits of agreement being between −0.20 and 0.22 L. The ICC between the manual and automated analyses was 0.96 (95% CI: 0.93–0.98), with a CV of 2.30 (95% CI: 1.87–2.73) %.

#### 3.3.3 Posterior Thigh Muscle Volume

When assessed manually, MRI‐derived posterior thigh muscle volume declined by 4% (185 ± 125 mL) following bed rest, from 4.3 ± 0.7 to 4.1 ± 0.7 L (*p* < 0.001; Figure 4E). When assessed automatically, MRI‐derived posterior thigh muscle volume also showed a decline by 4% (183 ± 71 mL) following bed rest, from 4.4 ± 0.7 to 4.2 ± 0.7 L (*p* < 0.001; Figure 4F). The magnitude of atrophy did not differ between the two methods (*p* = 0.984) and only marginally higher values (3.3 ± 4.6%) of posterior thigh muscle volume were observed with automated versus manual analyses for both the pre‐ and post‐bed rest values.

A very strong correlation was observed between the manual and automated determination of posterior thigh muscle volume (*r* ^*2*^ = 0.93, *r* = 0.96, 95% CI: 0.94 to 0.98, *p* < 0.001). In addition, a very good agreement was observed between both analysis methods, as assessed with the Bland–Altman plot, with a low bias (−0.12 ± 0.19 L) and 95% limits of agreement being between −0.49 and 0.24 L. The ICC between the manual and automated analyses was 0.95 (95% CI: 0.84–0.98) with a CV of 3.17 (95% CI: 2.56–3.79) %.

### 3.4 Correlations Between Imaging Techniques

Strong significant correlations were observed between MRI measurements (encompassing both manual and automated analysis methods) and those obtained from DXA and CT, as detailed in Table 1.

**TABLE 1:** Correlation of leg muscle mass assessments by different imaging techniques in healthy young males (*n* = 12), comparing measurements from both legs before and after a 2‐week bed rest period.

MRI‐analysis method	Imaging technique	*r*	95% CI	*p*
Manual (thigh muscle volume)	DXA (leg lean mass)	0.95	0.91–0.97	< 0.001
Automated (thigh muscle volume)	DXA (leg lean mass)	0.98	0.96–0.99	< 0.001
Manual (thigh muscle volume)	CT (thigh muscle CSA)	0.74	0.58–0.85	< 0.001
Automated (thigh muscle volume)	CT (thigh muscle CSA)	0.79	0.64–0.87	< 0.001

Abbreviations: CI, confidence interval; CSA, cross‐sectional area; CT, computed tomography; DXA, dual‐energy X‐ray absorptiometry; MRI, magnetic resonance imaging; *r*, Pearson correlation coefficient.

## 4 Discussion

In the present study, we observed that DXA, CT, and MRI all show a ∼5% decline in leg muscle quantity following 2 weeks of bed rest in healthy young men. When using MRI, disuse muscle atrophy can be accurately quantified using an automated approach, suggesting that automated methods could replace manual analysis in (many) clinical settings.

Imaging techniques are key for the accurate assessment of muscle atrophy. Most notable atrophy during a period of bed rest is generally seen in the lower body (Fuchs, Hermans, et al. 2024; LeBlanc et al. 1992), which compromises proper ambulation and overall mobility (Visser et al. 2005; Reid et al. 2008). Consequently, it is crucial to assess changes in leg muscle mass in a clinical setting. DXA is an imaging method that is commonly applied to estimate leg lean mass, serving as a proxy for muscle, and to monitor its changes over time in various settings (Mcglory et al. 2019; Edwards et al. 2020; Brook et al. 2022; Wall et al. 2014; Fuchs et al. 2023; Maden‐Wilkinson et al. 2013; Dirks et al. 2016; English et al. 2016). However, DXA measurements are sensitive to factors such as hydration status and food intake, and even in specific body regions such as the leg, its accuracy can be affected by these variables (Bone et al. 2017). To ensure reliable assessments, we carefully controlled these variables in this study. Our data confirm the efficacy of DXA in detecting small changes in leg lean mass following a 2‐week bed rest period in healthy individuals by showing a 5% decline in leg lean mass from 10.2 ± 1.6 to 9.7 ± 1.6 kg (Figure 2).

In contrast to DXA, CT directly images muscle tissue and is generally considered a more accurate method to assess (smaller) changes in leg muscle mass (Fuchs et al. 2023; Delmonico et al. 2008). Here, we also show the effectiveness of CT to assess disuse‐induced leg muscle atrophy, with a 6% decline in thigh muscle CSA from 155 ± 26 to 146 ± 24 cm^2^ following 2 weeks of bed rest (Figure 3). However, given the exposure to ionizing radiation, leg CT scans are often performed only on specific anatomical locations of the leg, thereby lacking the ability to assess changes in muscle form and size across the entire limb.

MRI overcomes many of the limitations of both DXA and CT and allows a more detailed evaluation of total muscle volume and mass (Fuchs et al. 2023). In this study, we also applied MRI to assess changes in leg muscle volume following the same 2‐week bed rest period. We observed a 5% decline in leg muscle volume from 7.1 ± 1.1 to 6.7 ± 1.0 L following bed rest (Figure 4A). The MRI data on the decline in leg muscle volume were strongly correlated with the data obtained from DXA and CT (*r* = 0.95 and *r* = 0.74, respectively; Table 1). In contrast to DXA and CT, MRI also offers the ability to quantify volumetric measurements of distinct muscle groups, as we (Fuchs, Hermans, et al. 2024; Fuchs et al. 2023; Kilroe et al. 2020; Fuchs, Trommelen, et al. 2024) and others (Bass et al. 2021; Belavý et al. 2009; Hogrel et al. 2015; Maden‐Wilkinson et al. 2013, 2014) have shown previously. In the present study, we assessed the impact of 2 weeks of bed rest on two separate (anterior and posterior) thigh muscle compartments (Figure 4C,E). We observed greater bed rest‐induced muscle atrophy (7%) in the anterior (primarily composed of the quadriceps femoris muscle group) compared to the posterior (4%) leg muscle compartment. This agrees with previous findings, showing that quadriceps muscle is more susceptible to disuse‐induced atrophy compared to other thigh muscle groups (Kilroe et al. 2020; Belavý et al. 2009). Clearly, MRI provides a substantial advantage in the comprehensive assessment of muscle tissue volume, offering a greater level of detail with regard to muscle atrophy than is feasible with the application of either DXA or CT in a clinical research setting.

In this study, the analysis of the entire thigh muscle volume, which includes differentiation between the anterior and posterior compartments, required an average of five full working days per individual for each time point assessed. The procedure involves detailed (digital) coloring of each 3 mm thick transverse/axial slice (Figure 1). The total MRI data analysis for all participants across all time points, including quality checks, required 3 months fulltime for 2 (independent) researchers. Consequently, this method is too time‐intensive and, as such, not practical and very costly. To address this issue, numerous companies and software platforms have developed and improved software to expedite analysis. In the present study, we used an automated analysis tool to analyze our muscle images and compare with our detailed manual analyses (Figure 1). When comparing these data, we found matching results for thigh muscle volume (Figure 4A vs. 4B), showing the same decline of 5% following bed rest, with only marginally (1.8 ± 4.1%) higher muscle volume values for automated versus manual analyses. This slight difference is likely due to random errors, as individual data points did not consistently show higher values with automated segmentation. A strong correlation (*r* = 0.96; Figure 5) and agreement (i.e., an ICC of 0.96 and a bias of −0.11 ± 0.29 L, with 95% limits of agreement between −0.67 and 0.45 L; Figure 6) with a small variation (CV: 2.74%) between the two analysis approaches were evident. This convinced us on the precision and reliability of automated MRI analysis in detecting subtle changes in muscle atrophy due to disuse. Also, when we performed a more detailed evaluation of specific (anterior and posterior) thigh muscle compartments, and compared this between the automated and our manual analysis, we also observed similar findings (Figure 4C vs. 4D & 4E vs. 4F). The automated MRI analysis seems to offer not only an accurate measure of the overall muscle volume but also facilitates the differentiation of individual muscle groups. The latter conclusions are also supported by robust correlations and agreements, validated through ICC analyses and Bland–Altman plots. However, while automated MRI is reliable for assessing larger areas, such as the entire thigh or thigh muscle groups, its accuracy for segmenting individual muscles within a muscle group and detecting disuse‐induced atrophy remains to be further refined and validated. Previous studies, assessing AMRA's methodology, have demonstrated low error margins, high test‐retest reliability, and a very strong correlation to the manual segmentation of leg muscle volume (Thomas et al. 2014). Further research also confirmed these findings (Middleton et al. 2017; West et al. 2018; Mandić et al. 2020), with one study demonstrating the method's capacity in detecting significant muscle volume changes following 8 weeks of resistance‐type exercise training (Mandić et al. 2020). Our findings extend on this body of work by demonstrating the effectiveness of automated MRI analysis in measuring muscle volume during short periods of whole‐body disuse, thereby showing that AMRA researcher can render laborious manual analysis redundant in such scenarios.

## 5 Conclusions

DXA, CT, and MRI all show a ∼5% decline in leg muscle quantity following 2 weeks of strict bed rest in healthy, male adults. MRI provides a more detailed view of muscle wasting, and the use of automated MRI analysis allows for accurate measurement of disuse muscle atrophy, rendering laborious manual analysis obsolete. Future studies should explore the capability of (other) automated methods to assess changes in individual muscles, especially as clinical interest in more precise details of muscle atrophy grows.

## Author Contributions

**Cas J. Fuchs:** conceptualization, methodology, formal analysis, investigation, writing – original draft preparation, funding acquisition. **Wesley J. H. Hermans:** methodology, formal analysis, investigation. **Job van den Hurk:** methodology, resources. **Christopher J. Wiggin:** methodology, resources. **Per Widholm:** methodology, formal analysis, investigation, resources. **Olof Dahlqvist Leinhard:** methodology, resources. **Pandichelvam Veeraiah:** methodology, resources. **Joachim E. Wildberger:** methodology, resources. **Jeanine J. Prompers:** methodology, resources. **Luc J. C. van Loon:** conceptualization, writing – review and editing, funding acquisition. All authors commented on previous versions of the manuscript and read and approved the final manuscript.

## Conflicts of Interest

Cas J. Fuchs and Luc J. C. van Loon collaborated and received research support from AMRA Medical AB. Per Widholm and Olof Dahlqvist Leinhard are employees of AMRA Medical AB. The other authors have no conflicts of interest related to this work.

## Data Availability

The datasets generated during and/or analyzed during the current study are available from the corresponding author on reasonable request.
